# Analysis of the Dynamic Characteristics of a Micro-Piezoelectric Bimorph Beam Based on an Admittance Test

**DOI:** 10.3390/mi8070220

**Published:** 2017-07-14

**Authors:** Tianxiang Zheng, Shuo Chen, Linxu Lei, Zhanfeng Deng, Cheng Zhang, Xing Yang, Haodong Zou, Menghan Xu

**Affiliations:** 1 State Key Laboratory of Advanced Power Transmission Technology, Global Energy Interconnection Research Institute, State Grid Corporation of China, Beijing 102200, China; zhengtianxiang@geiri.sgcc.com.cn (T.Z.); chenshuo@geiri.sgcc.com.cn (S.C.); leilinxu@geiri.sgcc.com.cn (L.L.); dengzhanfeng@geiri.sgcc.com.cn (Z.D.); 2 The State Key Laboratory of Precision Measurement Technology and Instrumentation, Tsinghua University, Beijing 100084, China; zhangchengthu@163.com; 3 Key Laboratory of Special Purpose Equipment and Advanced Manufacturing Technology, Ministry of Education, Zhejiang University of Technology, Hangzhou 310014, China; 4 Information & Telecommunication Branch, State Grid Jiangsu Electric Power Company, State Grid Corporation of China, Nanjing 210024, China; haodongzou@163.com (H.Z.); xumenghan16@126.com (M.X.)

**Keywords:** microelectromechanical systems (MEMS), bimorph beams, dynamic characteristics, resonant frequency, equivalent circuit, admittance

## Abstract

A piezoelectric bimorph beam, as an upgraded cantilever beam structure, can be used to detect gas content and build a micro-actuator, among other functions. Thus, this beam is widely applied to microelectromechanical systems (MEMS), transformers, and precision machinery. For example, when photoacoustic spectroscopy is performed to detect oil-soluble gas in transformers, a micro-cantilever beam can be used to detect gas content. The dynamic characteristics of piezoelectric bimorph beams, such as resonant frequency, are important indexes in the applications of these beams. The equivalent circuit model for a piezoelectric bimorph beam is examined in this study and an admittance test is performed on the beam to accurately, quickly, and economically measure and analyze its dynamic characteristics. Then, the least squares method is applied to obtain the characteristic curves of the admittance circle, amplitude frequency, and phase frequency; identify the dynamic characteristics of the piezoelectric bimorph beam (e.g., resonant frequency); and determine the parameters of the equivalent circuit. The resonant frequency of the piezoelectric bimorph beam is 207.67 Hz based on the result of the admittance circle test, which is basically consistent with the results of microscope image method (i.e., 207.85 Hz) and the theoretical calculation (i.e., 222.03 Hz). This finding proves the validity of the proposed test method. This method cannot only improve the detection speed of piezoelectric bimorph beams, but can also provide a fast detection strategy for testing the characteristics of such beams during photoacoustic spectroscopy.

## 1. Introduction

A micro-piezoelectric bimorph beam is made of piezoelectric, elastic, and metallic materials, with the metallic materials acting as a common electrode [1,2,3]. This type of beam can effectively amplify the static displacement and amplitude of piezoelectric materials, and thus, it is widely used in transformers, microelectromechanical systems (MEMS), and precision machinery, among other functions [4,5]. For example, the detection of an oil-soluble gas in transformers is a critical monitoring procedure that can effectively reflect overheating and partial discharge in transformers. The detection process is described as follows. An oil and gas separation system is used to extract trace gas dissolved in transformer insulation oil. Partial overheating and discharge level within the transformer is then determined based on gas content. Subsequently, the components of the gas mixture are identified and separated via photoacoustic spectroscopy to conduct a comprehensive assessment of transformer performance. A micro-piezoelectric bimorph beam can be used as the sound signal detection device in photoacoustic spectrum detection [6,7,8].

The dynamic characteristics of piezoelectric bimorph beams, such as resonant frequency, are important indexes in the applications of such beams [9,10,11]. At present, laser Doppler, stroboscopic micro-vision, and stroboscopic micro-interference can achieve dynamic characteristics for a high-precision, non-contact measurement of microstructures [12,13]. However, these test methods exhibit low efficiency and require complex equipment. Therefore, an admittance test method is proposed in this study to realize low-cost, accurate, and fast measurement and analysis of the dynamic characteristics of piezoelectric bimorph beams. This method is based on the electromechanical coupling characteristics of these beams. The mechanical vibration characteristics of a piezoelectric bimorph beam can be determined by measuring and analyzing its electrical admittance characteristics [14,15].

## 2. Analysis of the Equivalent Circuit and Principle of a Piezoelectric Bimorph Beam

**Figure 1:**
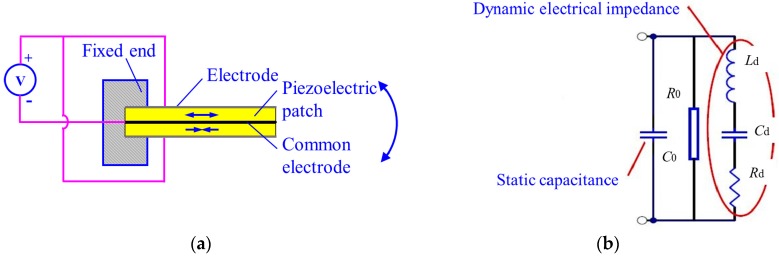
Piezoelectric bimorph beam (**a**) the structure; (**b**) equivalent circuit.

The structure of the commonly used piezoelectric bimorph beam is shown in Figure 1a. This beam is composed of two piezoelectric patches with the same polarization directions and a common electrode in the middle. The two piezoelectric patches are wired, and alternating current (AC) voltage is applied (Figure 1a). The driving electric fields applied to the plates are always opposite each other. The polarization directions of the two piezoelectric patches are the same; hence, one of the plates contracts, whereas the other expands. This condition causes the piezoelectric bimorph beam to bend and swing. The swing frequency corresponds to the applied alternating current frequency.

A piezoelectric bimorph beam is an electromechanical coupling device. Its equivalent circuit is shown in Figure 1b [16], where *C*_0_ is the static capacitance, *R*_0_ is the dielectric loss parallel resistance, *R_d_* is the dynamic resistance, *C_d_* is the parallel capacitance, and *L_d_* is the dynamic inductance. When the mechanical quality factor of a component is high, *C_d_* and *L_d_* can be considered constant within the range of a given resonance frequency. The value of *R_d_* is related to the mechanical energy of mechanical loss. The impedance of the circuit is simplified as its static admittance as

(1) $$Y_{0}=\frac{1}{R_{0}}+i\omega C_{0}=G_{0}+iB_{0}$$

Dynamic admittance:

(2) $$Y_{d}=\frac{1}{R_{d}+i(\omega L_{d}-\frac{1}{\omega C_{d}})}=G_{d}+iB_{d}$$

Total admittance:

(3) $$Y=Y_{0}+Y_{d}=G+iB$$

The solid and dotted lines of the dynamic admittance on the complex plane form a circle, which is called an admittance circle.

(4) $${\left[G_{d}-\frac{1}{2R_{d}}\right]}^{2}+{B_{d}}^{2}={\left(\frac{1}{2R_{d}}\right)}^{2}$$

Similarly, the total admittance on the complex plane also forms an admittance circle.

(5) $${\left[G-\left(\frac{1}{2R_{d}}+\frac{1}{R_{0}}\right)\right]}^{2}+{\left(B-\omega C_{0}\right)}^{2}={\left(\frac{1}{2R_{d}}\right)}^{2}$$

**Figure 2:**
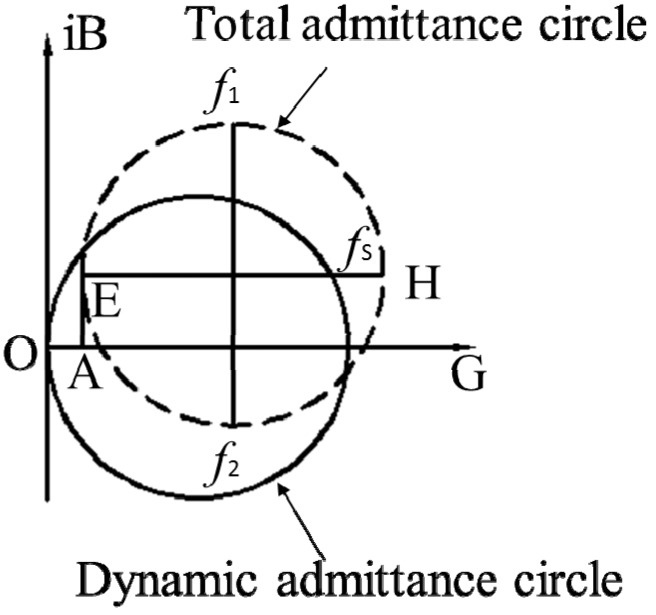
Dynamic admittance circle and total admittance circle.

The dynamic admittance circle and the total admittance circle are represented by solid and dotted lines, respectively, in Figure 2.

The diagram of the admittance circle is used to identify all the parameters of the equivalent circuit of the piezoelectric bimorph beam. The diameter of the circle is set as *D*, then

(6) $$R_{d}=\frac{1}{D}$$

A straight line (EH) is drawn parallel to the conductive axis that passes through the center of the circle. Then, the circle is intersected at point H. The frequency that corresponds to point H is the mechanical resonance frequency *f_s_*. Static capacitance and dielectric loss resistance can then be determined.

Using the corresponding distance $\bar{AE}$ from Figure 2, the static capacitance can be determined

(7) $$C_{0}=\frac{\bar{AE}}{2\pi f_{s}}$$

Using the corresponding distance $\bar{OA}$ from Figure 2, the dielectric loss resistance can be determined

(8) $$R_{0}=\frac{1}{\bar{OA}}$$

A straight line is drawn perpendicular to the conductive axis. The intersection frequencies are *f*_1_ and *f*_2_ [17,18,19]. Then, using the corresponding distances and frequencies from Figure 2, the dynamic inductance and parallel capacitance can be determined.

Dynamic inductance:

(9) $$L_{d}=\frac{R_{d}}{2\pi f_{2}-2\pi f_{1}}$$

Parallel capacitance:

(10) $$C_{d}=\frac{1}{{\left(2\pi f_{s}\right)}^{2}L_{d}}$$

## 3. Methods for the Admittance Test of a Piezoelectric Bimorph Beam

**Figure 3:**
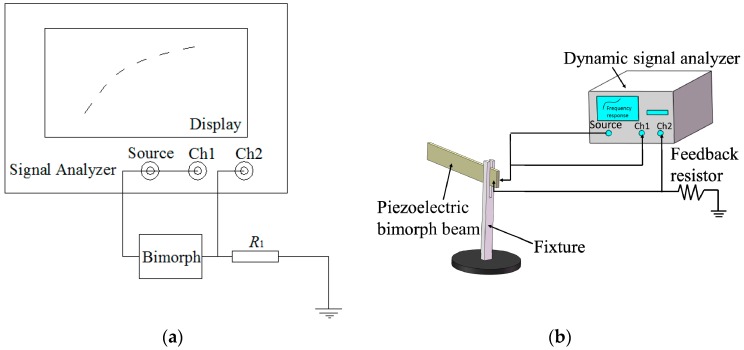
The admittance test of a piezoelectric bimorph beam. (**a**) The detection principle; (**b**) Schematic diagram of the admittance test system.

This study uses a dynamic signal analyzer to test the admittance spectrum of a piezoelectric bimorph beam based on the preceding theoretical analysis. The detection principle is illustrated in Figure 3a. In the figure, the piezoelectric bimorph beam and feedback resistance *R*_1_ (resistance value = 1 Ω; hence, the values of the passing voltage and current are equal) are in series connection: one end is connected to the ground, whereas the other end is connected to the signal source of the dynamic signal analyzer. The voltage value *U*_1_ of the signal source and the voltage value *U*_2_ of the feedback resistor are input into Channel 1 (Ch1) and Channel 2 (Ch2) of the analyzer, respectively. The function of the feedback resistor is to convert the current signal of the piezoelectric bimorph beam into a voltage signal for the input analyzer. The calculation function of the dynamic signal analyzer can be used to obtain the admittance *Y*_c_(ω) (reverse of resistance) of the piezoelectric bimorph beam as

(11) $$Y_{c}(\omega )=\frac{\vec{I}}{{\vec{U}}_{c}}\approx \frac{{\vec{U}}_{1}}{\vec{U}}$$

where $\vec{I}$ and ${\vec{U}}_{c}$ are the current and voltage of the piezoelectric bimorph beam, respectively; and ${\vec{U}}_{1}$ and $\vec{U}$ are the feedback resistor and voltage of the signal source, respectively.

As shown in Figure 3b, the admittance test system for the piezoelectric bimorph beam comprises the piezoelectric bimorph beam, a fixture, a feedback resistor, and a dynamic signal analyzer (SR785, Stanford Research Systems, Inc., Sunnyvale, CA, USA). The piezoelectric bimorph beam is 50 mm long, 2 mm wide, and 0.7 mm thick. The fixture is used to clamp one end of the piezoelectric bimorph beam, and the cantilever beam is left with a length of 35 mm. The dynamic signal analyzer is used to apply excitation signals with different frequencies to the piezoelectric bimorph beam to measure the admittance value of the beam and obtain the admittance circle. Consequently, the resonant characteristics of the piezoelectric bimorph beam can be analyzed.

## 4. Experiment Result and Discussion of the Admittance Test

**Figure 4:**
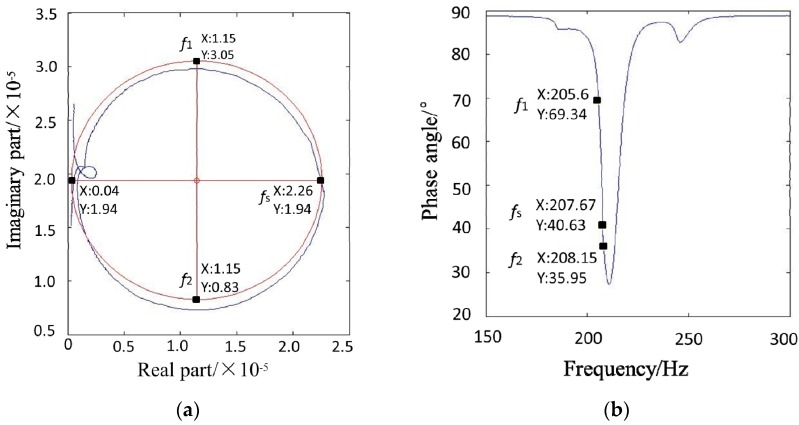
(**a**) Admittance circle curve; (**b**) Phase frequency characteristic curve.

The admittance test system for the piezoelectric bimorph beam is used to measure the admittance circle curve (blue line in Figure 4a) and the phase frequency characteristic curve (Figure 4b). As shown in Figure 4a, the measured admittance curve is not perfectly round due to various interference factors, such as interference curves. To determine the relevant parameters, this study uses the circular fitting method to make the fitted circle (red line in Figure 4a) an approximation of the measured admittance circle through MATLAB software (R2010a, MathWorks, Inc., Natick, MA, USA). The specific method is described as follows [20].

The center of the admittance circle is set as (−*a*/2, −*b*/2), the radius is *r*, and the equation of the circle is

(12) $${\left(x+\frac{a}{2}\right)}^{2}+{\left(y+\frac{b}{2}\right)}^{2}=r^{2}$$

That is,

(13) $$\begin{array}{r} x^{2}+y^{2}+ax+by+c=0\\ c={\left(\frac{a}{2}\right)}^{2}+{\left(\frac{b}{2}\right)}^{2}-r^{2} \end{array}\}$$

Through the least squares method,

(14) $$\left[\begin{array}{l} a\\ b\\ c \end{array}\right]=\left[\begin{array}{ccc} \sum_{i=1}^{m}{x_{i}}^{2} & \sum_{i=1}^{m}x_{i}y_{i} & \sum_{i=1}^{m}x_{i}\\ \sum_{i=1}^{m}x_{i}y_{i} & \sum_{i=1}^{m}{y_{i}}^{2} & \sum_{i=1}^{m}y_{i}\\ \sum_{i=1}^{m}x_{i} & \sum_{i=1}^{m}y_{i} & m \end{array}\right]\left[\begin{array}{l} -\sum_{i=1}^{m}\left({x_{i}}^{3}+x_{i}{y_{i}}^{2}\right)\\ -\sum_{i=1}^{m}({x_{i}}^{2}y_{i}+{y_{i}}^{3})\;\\ -\sum_{i=1}^{m}\left({x_{i}}^{2}+{y_{i}}^{2}\right) \end{array}\right]$$

where (*x_i_*, *y_i_*) are the coordinates at a certain point on the measured admittance circle.

The center of the fitted admittance circle is (1.15 × 10^−5^, 1.94 × 10^−5^) and the radius is 1.11 × 10^−5^ m, which can determine the coordinates of the frequency points of the piezoelectric bimorph beam on the admittance circle curve (Figure 4a), the resonant frequency point *f_s_* (2.26 × 10^−5^, 1.94 × 10^−5^), and the two auxiliary frequency points, i.e., *f*_1_ (1.15 × 10^−5^, 3.05 × 10^−5^) and *f*_2_ (1.15 × 10^−5^, 8.3 × 10^−6^). The coordinates of each frequency point are used to obtain their phase angle *θ* according to the formula *θ* = arctan(*Y*/*X*). The frequency region of the phase angle in the phase frequency characteristic curve is observed. Then, MATLAB software is used to search for the coordinates of each frequency point in the phase frequency characteristic curve (Figure 4b), the resonant frequency point *f_s_* (207.67, 40.63), the auxiliary frequency point *f*_1_ (205.60, 69.34), and the auxiliary frequency point *f*_2_ (208.15, 35.95) is obtained. Therefore, the resonant frequency point *f_s_* = 207.67 Hz and the auxiliary frequency points *f*_1_ = 205.60 Hz, *f*_2_ = 208.15 Hz are determined.

Subsequently, all the parameters in the equivalent circuit of the piezoelectric bimorph beam are obtained as

$$R_{d}=\frac{1}{D}=\;4.50\;\times \;10^{4},\;L_{d}=\frac{R_{d}}{2\pi f_{2}-2\pi f_{1}}=\;2810.04\;H,\;C_{d}=\frac{1}{{\left(2\pi f_{s}\right)}^{2}L_{d}}=\;2.09\;\times \;10^{-10}\;F,\;C_{0}=\frac{\bar{AE}}{2\pi f_{s}}=\;8.82\;\times \;10^{-9}\;F,\;\text{and}\;R_{0}=\frac{1}{\bar{OA}}=\;2.93\;\times \;10^{6}\;\Omega .$$

**Figure 5:**
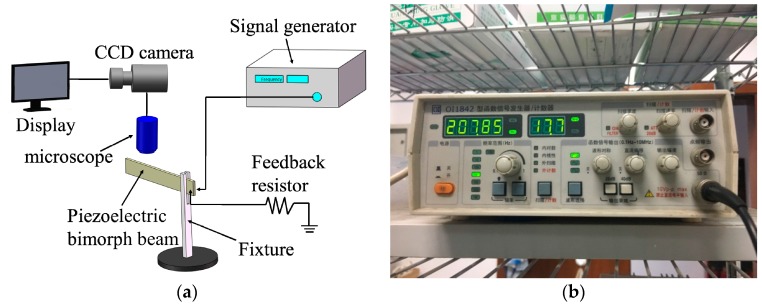
(**a**) Schematic diagram of the microscope image observation method and (**b**) the value of the signal generator when the piezoelectric bimorph beam is in a resonant state.

To verify the feasibility and accuracy of the admittance test result, the microscope image observation method is used to retest the resonant frequency of the piezoelectric bimorph beam. A system diagram of the microscope image observation method is shown in Figure 5a. The system includes the piezoelectric bimorph beam, a fixture, a signal generator (OI1842, Beijing Ocean Xingye Technology Co., Ltd., Beijing, China), and a probe system (M150, Cascade Microtech, Inc., Beaverton, OR, USA); and the probe system contains a microscope, a charge-coupled device (CCD) camera, and a display. The principle of the test is described as follows. When the signal generator provides electrical signals to the piezoelectric bimorph beam, the beam begins to vibrate. The vibration of the beam is observed through the microscope and the CCD camera. The piezoelectric bimorph beam is in a resonant state when the frequency of the electrical signals supplied to it is equal to its resonant frequency. The amplitude of the piezoelectric bimorph beam observed on the display is highest during that time, and the excitation signal frequency displayed on the signal generator is the resonant frequency of the beam.

To ensure the accuracy of the test experiment, we use the same clamping method applied to the admittance test—i.e., the fixture is used to clamp one end of the piezoelectric bimorph beam—while leaving the cantilever beam with a length of 35 mm. Thereafter, the signal generator is used to apply excitation signals with different frequencies to the piezoelectric bimorph beam. The vibration of the piezoelectric bimorph beam is observed on the display.

The value of the signal generator is shown in Figure 5b when the amplitude of the piezoelectric bimorph beam is at its highest during the experiment. The piezoelectric bimorph beam is in a resonant state when the electrical signal is 207.85 Hz. Therefore, the resonant frequency is 207.85 Hz, which is basically consistent with the resonant frequency of 207.67 Hz obtained through the admittance circle. This result proves the feasibility and accuracy of applying the admittance circle to analyze the dynamic characteristics of a piezoelectric bimorph beam.

Moreover, the resonant frequencies of the piezoelectric bimorph beam can be theoretically calculated. We have calculated the resonant frequency of the piezoelectric bimorph beam as 222.03 Hz, when compared to the resonant frequencies obtained from the experiments, the differences are less than 8%, and the differences may be caused by the measurement errors, including measure of the cantilever beam size, the clamping position of the cantilever beam, and so on. Therefore, the theoretically calculated result can also prove the feasibility and accuracy of applying the admittance circle to analyze the dynamic characteristics of a piezoelectric bimorph beam.

## 5. Results

In summary, this study presents the application of an admittance test to determine the dynamic characteristics of a piezoelectric bimorph beam. A dynamic signal analyzer is used to perform the admittance test on the beam. The admittance circle and phase frequency curves of the piezoelectric bimorph beam are fitted by using MATLAB software. Then, the dynamic characteristics of the piezoelectric bimorph beam, such as resonant frequency, are obtained by analyzing the admittance circle and phase frequency curves, and then the equivalent circuit parameters are identified. Finally, the microscopic image observation method and theoretical calculation are conducted to redetermine the resonant frequency of the piezoelectric bimorph beam. The results are basically the same as the result of the admittance test, thereby proving the feasibility and accuracy of applying the admittance circle to analyze the dynamic characteristics of a piezoelectric bimorph beam. Compared with using laser Doppler, stroboscopic micro-vision, and stroboscopic micro-interference to measure the dynamic characteristics of microstructures, the instruments required for the proposed test method are relatively simple, low-cost, and highly efficient. Thus, the proposed method has a high application value.
